# Assessing the potential of translocating vulnerable forest birds by searching for novel and enduring climatic ranges

**DOI:** 10.1002/ece3.3451

**Published:** 2017-09-27

**Authors:** Lucas B. Fortini, Lauren R. Kaiser, Adam E. Vorsino, Eben H. Paxton, James D. Jacobi

**Affiliations:** ^1^ Pacific Island Ecosystems Research Center US Geological Survey Honolulu HI USA; ^2^ Pacific Islands Climate Change Cooperative Honolulu HI USA; ^3^ University of Hawai'i at Mānoa Honolulu HI USA; ^4^ Strategic Habitat Conservation Division Pacific Islands Office US Fish & Wildlife Service Honolulu HI USA

**Keywords:** assisted colonization, assisted migration, avian malaria, climate vulnerability, niche overlap, species distribution model

## Abstract

Hawaiian forest birds are imperiled, with fewer than half the original >40 species remaining extant. Recent studies document ongoing rapid population decline and project complete climate‐based range losses for the critically endangered Kaua'i endemics ‘akeke’e (*Loxops caeruleirostris*) and ‘akikiki (*Oreomystis bairdi*) by end‐of‐century due to projected warming. Climate change facilitates the upward expansion of avian malaria into native high elevation forests where disease was historically absent. While intensified conservation efforts attempt to safeguard these species and their habitats, the magnitude of potential loss and the urgency of this situation require all conservation options to be seriously considered. One option for Kaua’i endemics is translocation to islands with higher elevation habitats. We explored the feasibility of interisland translocation by projecting baseline and future climate‐based ranges of ‘akeke’e and ‘akikiki across the Hawaiian archipelago. For islands where compatible climates for these species were projected to endure through end‐of‐century, an additional climatic niche overlap analysis compares the spatial overlap between Kaua’i endemics and current native species on prospective destination islands. Suitable climate‐based ranges exist on Maui and Hawai'i for these Kaua'i endemics that offer climatically distinct areas compared to niche distributions of destination island endemics. While we recognize that any decision to translocate birds will include assessing numerous additional social, political, and biological factors, our focus on locations of enduring and ecologically compatible climate‐based ranges represents the first step to evaluate this potential conservation option. Our approach considering baseline and future distributions of species with climatic niche overlap metrics to identify undesirable range overlap provides a method that can be utilized for other climate‐vulnerable species with disjointed compatible environments beyond their native range.

## 1 INTRODUCTION

The risk of extinction from climate change is predicted to increase and intensify with rising temperatures, threatening one in six species in the future (Urban, 2015). Fragmented landscapes, either natural or anthropogenic, limit the dispersal of species to potential suitable habitats, further exacerbating the risk of extinction, especially for endemic species with small ranges (Williams, Jackson, & Kutzbach, 2007). Conservation within a shifting climate context for at‐risk species should be multifaceted and combine many standard and innovative actions which minimize both nonclimatic and climatic threats (Fortini, Vorsino, Amidon, Paxton, & Jacobi, 2015). Given the concurrent rates of climate change and extinctions, contemporary conservation efforts including translocation strategies, bioengineering adaptations, captive preservation of populations, and other options (Heller & Zavaleta, 2009; Mawdsley, O'malley, & Ojima, 2009) need to be seriously evaluated for potential implementation.

Translocation of species is beginning to be considered as an option for direct response to pressures of climate change (Van der Veken et al., 2012; Willis et al., 2009). Most studies on this topic that used applied modeling approaches to prioritize efforts for translocation focused on the reestablishment of species within historic ranges (Carroll et al., 2009; Freifeld, Plentovich, Farmer, & Wallace, 2012; Martínez‐Meyer, Peterson, Servin, & Kiff, 2006; Pearce & Lindenmayer, 1998; Reynolds, Weiser, Jamieson, & Hatfield, 2013; Vitt, Havens, & Hoegh‐Guldberg, 2009). A few others have documented the use of translocation as a conservation tool to establish populations in new areas beyond a species' historic range (Dade, Pauli, & Mitchell, 2014; Derrickson, Beissinger, & Snyder, 1998; Laws & Kesler, 2012). However, translocation‐based conservation strategies, especially beyond a species historic range, are controversial (Armstrong, Hayward, Moro, & Seddon, 2015; Lunt et al., 2013; Seddon, 2010). There are valid arguments both for (Hoegh‐Guldberg et al. 2008) and against (Ricciardi and Simberloff 2009) translocation. Ecological arguments opposing this approach contend that identifying suitable locations outside a species' historic range is impossible (Davidson & Simkanin, 2008) and that they may be less suitable or lead to an invasive species problem (Huang, 2008), while others argue that translocations are necessary in some cases to save species from extinction (Hoegh‐Guldberg et al., 2008; McLachlan, Hellmann, & Schwartz, 2007; Thomas, 2011; Vitt et al., 2009).

Hawaiian native forest birds have been a major focus of conservation efforts in Hawai'i (Camp, Gorresen, Pratt, & Woodworth, 2009; Pratt, 1994). This species group has experienced multiple extinctions (Gorresen, Camp, Reynolds, Woodworth, & Pratt, 2009; Ohlemuller et al., 2008) and today is mostly absent from lower elevations primarily due to habitat loss and the introduction of avian malaria and its mosquito vector (Atkinson et al., 2014; Behnke, Pejchar, & Crampton, 2015; Benning, LaPointe, Atkinson, & Vitousek, 2002; van Riper & Scott, 2001). Hawai'i's sharp climatic gradients have allowed the native bird species to persist in higher elevation areas where avian malaria cannot develop and mosquito densities are low (Benning et al., 2002; van Riper, van Riper, Goff, & Laird, 1986). Unfortunately, ongoing and projected climatic shifts threaten these remaining mosquito and disease‐free refugia. Mean temperatures have recently risen regionally by 0.163°C per decade over the last three decades (Giambelluca, Diaz, & Luke, 2008). This warming trend is even more pronounced at higher elevations (Diaz, Giambelluca, & Eischeid, 2011). Declining precipitation has also been observed, especially during the wet season (Chu & Chen, 2005; Giambelluca et al., 2013). Parallel to these climatic trends, recent research has documented substantial range contractions of all native forest birds on Kaua'i over the last four decades, with species losing 25%–70% of their range (Paxton et al., 2016). Given that both temperature and precipitation delineate the distribution of the mosquito vector of avian malaria and consequently the disease‐susceptible native forest birds (Ahumada, LaPointe, & Samuel, 2004; Benning et al., 2002; Liao et al., 2015), future changes in these environmental variables will likely further impact Hawaiian forest birds (Atkinson et al., 2014). In fact, extensive modeling efforts using downscaled end‐of‐century climate projections estimate a range loss of 50%–100% for most Hawaiian forest birds in the absence of effective vector control or increased disease resistance (Fortini et al., 2015).

Within this group of species that are extremely vulnerable to climate change, the Kaua'i endemics ‘akeke’e (*Loxops caeruleirostris*) and ‘akikiki, (*Oreomystis bairdi*) stand out. Both of these forest bird species have been deemed “Critically Endangered” owing to their rapid decline in population size over the last 10 years and the extremely small declining range available to these species on the island of Kaua’i (IUCN, 2016a). Current decline in their ranges has occurred more rapidly since 2000, limiting these two species to between 40 and 64 km^2^ (Paxton et al., 2016). Models of their future projected distributions consistently predict complete range losses by end‐of‐century (Fortini et al., 2015). Given that the island of Kaua'i itself offers no higher elevation habitat, persistence of these Kaua'i endemic species will require consideration of conservation options beyond ongoing efforts to manage their current habitat (Fortini et al., 2015). The severity of this situation compelled the recent 2016 International Union for the Conservation of Nature (IUCN) World Conservation Congress to approve a motion to support increased conservation efforts for Hawai'i's threatened birds (IUCN, 2016b). This motion calls for the deployment of various techniques that benefit conservation, specifically mentioning translocation, as extinction of these forest birds may be imminent without significantly expanding conservation efforts (IUCN, 2016b).

Using modeling methods for site selection to determine appropriate areas for translocated populations is an important initial step that can be used to develop strategies for establishing new sustainable and viable populations of species that are vulnerable to extinction (Chauvenet, Ewen, Armstrong, & Pettorelli, 2013; Rout et al., 2013). Our study provides a means to address a first critical issue that precedes any in‐depth consideration of possible novel species interactions caused by translocation. Namely, is translocation a feasible option to consider given the differences in climatic niche of species? To do this, we focused on answering two related questions: (i) Is there compatible climate‐based range for these translocation candidate species? If so, (ii) Is there potential for overlap with other species of concern that warrant further research into novel species interactions? Expanding on previous research (Fortini et al., 2015), we first project the baseline distribution of our two candidate translocation species, ‘akeke’e and ‘akikiki. By then modeling the potential range for these species across the entire archipelago under baseline and future climates, we attempt to identify areas outside the species' historic range that are likely to remain climatically suitable for the species through end‐of‐century. We then perform a climatic niche overlap analysis (Warren, Glor, & Turelli, 2008) to assess the potential for climate‐based range overlap among candidate species for translocation and resident island species under baseline and future climate scenarios. Our main focus is to identify and map locations of ecologically compatible climate‐based ranges for these two species. This combination of species distribution model (SDM) projections of baseline and future distributions, along with niche overlap analyses, offers a new toolset that provides foundational information necessary to species and location specific evaluation of the utility of translocation.

## 2 METHODS

### 2.1 Species surveys

**Figure 1:**
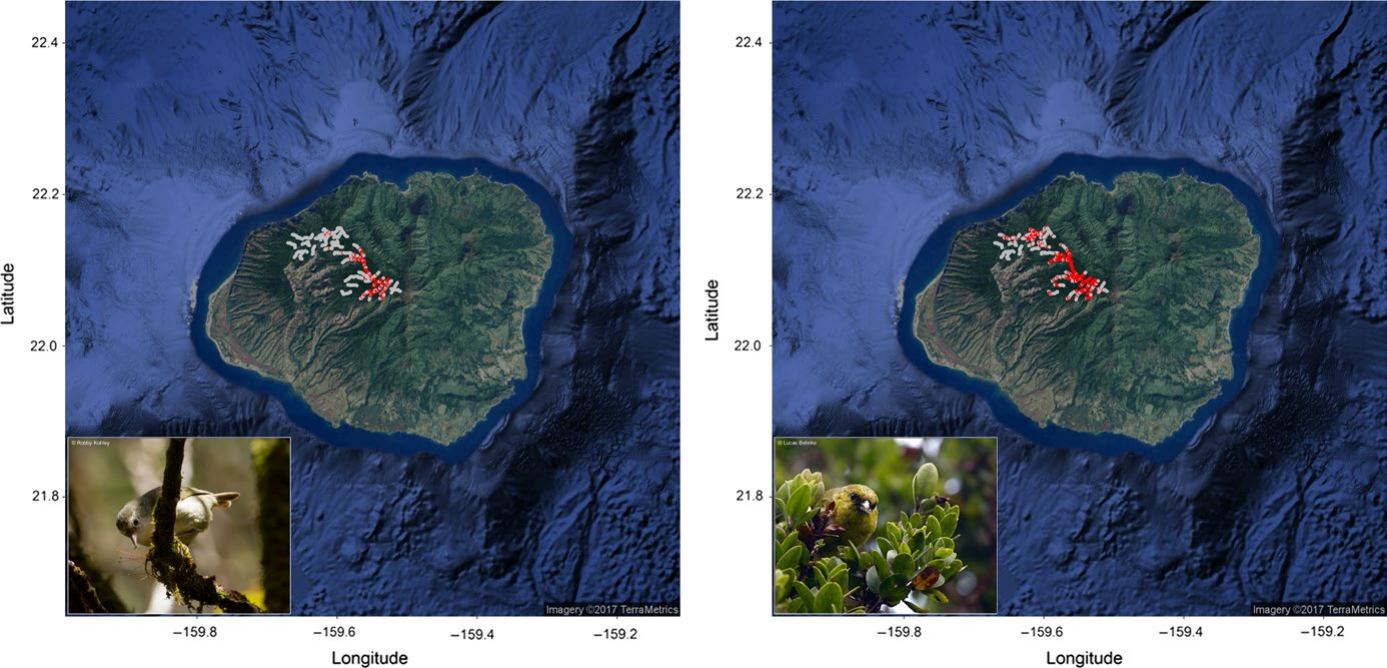
Point location data for Kaua'i endemics ‘akeke’e (*Loxops caeruleirostris*) on the left panel and ‘akikiki (*Oreomystis bairdi*) on the right panel. The red points show presence locations from observational surveys and the grey points show the surveyed absence data. Both species are rare and declining in their native habitat on Kaua’i. ‘Akeke’e feeds on the crown foliage of ‘ōhi’a trees by using its crossed bill to force open leaf buds in search of insects (Source: Photo © Lucas Behnke). ‘Akikiki, also known as the Kaua’i Creeper, forages for insects while creeping over tree trunks and along branches (Source: Photo © Robby Kohley)

Statewide surveys of bird populations collected since 1970 and archived in the Hawai'i Forest Bird Interagency Database (Pratt, Camp, & Gorresen, 2006) provided point location data for native Hawaiian forest birds. To further improve and gap fill this database, additional site‐ or species‐specific surveys were included (Camp, Pratt, Gorresen, Jeffrey, & Woodworth, 2010; Camp et al., 2009; Jacobi, Fancy, Giffin, & Scott, 1996; Vanderwerf, Lohr, Titmus, Taylor, & Burt, 2013; Vanderwerf, Rohrer, Smith, & Burt, 2001). Point location data for all native Hawaiian forest birds vary anywhere from about 50 to over 1,000 occurrence records depending on the species surveyed as well as which island(s) the species inhabits. A total of 239 and 111 presence records were available specifically for ‘akeke’e and ‘akikiki, respectively, on Kaua’i (Figure 1). Significant effort has been spent over the years to monitor these birds, and these numbers of presence records mainly reflect the rarity of these two species. As point‐count survey data provide limited absence data spread across the landscape due to the nature of observational surveys, pseudo‐absence points were randomly produced. Pseudo‐absences were randomized at an island‐specific average density of 1 per 3.125 km^2^ to account for differences of analysis extent between islands and were at least 500 m from any known presence location to yield stable model results while minimizing model computations (Fortini et al., 2015). The combined presence data with pseudo‐absence points created the complete dataset used for SDM projections.

### 2.2 Environmental predictors

Predictors selected for this analysis reflect the mean and variance of temperature and rainfall, both of which are related to avian malaria and forest bird ranges (Ahumada et al., 2004; Benning et al., 2002). The methodology for variable selection follows that of Fortini et al. (2015) to minimize multicollinearity. The four bioclimatic variables selected as predictors for the SDMs include mean annual temperature (Bio1), temperature annual range (Bio7), mean annual precipitation (Bio12), and precipitation seasonality (Bio15). These abiotic indices were defined for the main six Hawaiian Islands (Kaua'i, O'ahu, Moloka'i, Maui, Lana'i, and Hawai'i) from 250 m spatial resolution monthly rainfall averages (Giambelluca et al., 2013) and monthly minimum and maximum temperature averages (Daly, Conklin, & Unsworth, 2010), which were then aggregated up to 500 m to improve computing time for model projections. Baseline indices from 1990 to 2010 were calculated from the monthly temperature and precipitation data using the R package “dismo” (Hijmans, Phillips, Leathwick, & Elith, 2015). Future predictors from 2080 to 2100 were derived from the Hawaiian Regional Climate Model projections (Zhang, Wang, Lauer, & Hamilton, 2012), using the Special Report on Emissions Scenario (SRES) A1B which projects warmer and wetter future climatic conditions for Hawai'i. To evaluate the importance of future scenario uncertainty, we ran simplified species distribution models using only two bioclimatic variables (Bio1 and Bio12) to compare our SRES A1B‐based SDMs with SDMs based on only recently available end‐of‐century mean annual temperature and precipitation climate projections for representative concentration pathways (RCPs) 4.5 and 8.5 (Timm & Diaz, 2009). RCPs 4.5 and 8.5 have mean projected warming by end‐of‐century below and above (respectively) our main emission scenario considered (SRES A1B), but differences in downscaling approach tend to project considerably less rainfall for several parts of the archipelago using the statistically downscaled RCP projections when compared to the dynamically downscaled wetter SRES A1B projections.

### 2.3 Species distribution model projection

We first fit SDMs for ‘akeke’e and ‘akikiki using baseline (from 1990 through 2009) climate predictors from Kaua’i only. We then projected their distributions archipelago‐wide using the species point data and the same environmental predictors to determine new possible climate‐based ranges under baseline and future projected climates. The modeled species distributions were further refined by cropping them to areas within currently compatible vegetation based on a recent statewide vegetation map (Fortini et al., 2015; Rollins, 2009). By limiting the available range to compatible vegetation, estimated distributions are not projected to unsuitable habitat areas (e.g., bare lava flows, urban areas, high elevations). Shifts in current habitat vegetation cover were not considered to be a limiting factor given that such land cover changes would occur over a longer period of time in comparison with the more rapid ongoing impacts of climatic change. We applied a threshold derived from habitat suitability scores for modeled presence, based on equivalent sensitivity/specificity from the model evaluation data (Jiménez‐Valverde & Lobo, 2007; Liu, White, & Newell, 2011). This simplifies our interpreted model outputs to more easily make comparisons among species. For all SDMs, we used an ensemble modeling approach as it generally creates projections with better predictive ability (Thuiller, Engler, 2014). We performed a total of 220 model runs for each emissions scenario per species, using 80% of the data for training and 20% for testing. The ensemble models included generalized boosted models (GBM) and maximum entropy (MaxEnt) submodels based on their known predictive accuracy. GBM methods focus on classification trees that learn and improve on the accuracy of predictions through additive boosting of decision trees. MaxEnt employs a maximum entropy method comparing the projected distribution of location points to a null distribution based on pseudo‐absences to model the distribution of a species.

Receiver operating characteristic (ROC) scores were used to evaluate the performance and validity of all models and range from 0 to 1 where 0.5 indicates that a model's utility is as good as random with no skill distinguishing between two alternative events. Calculated mean ROC scores for Kaua'i, Maui, and Hawai'i native species are 0.98, 0.94, and 0.93, respectively. Archipelago‐wide, the mean ROC score is 0.98 across all SDMs (Table S1). Models with ROC scores <0.5 were excluded from the ensemble model building process to ensure the quality of our ensemble predictions and reduce uncertainty. Our simpler SDMs based only on Bio1 and Bio12 show nearly equivalent ROC scores for Kaua'i species, meaning they are suitable for our emission scenario comparisons. This applied modeling approach produced highly accurate baseline climate‐based species distributions that represent known geographic patterns identified by expert‐derived range maps (Fortini et al., 2015). Using the “biomod2” R package (Thuiller, Engler, 2014), all multimodel ensemble modeling, calibration, forecasting, and statistical analyses were performed iteratively. The R scripts used at time of publication along with test data are available online (https://doi.org/10.5066/F7NZ86K9).

### 2.4 Model transferability

**Figure 2:**
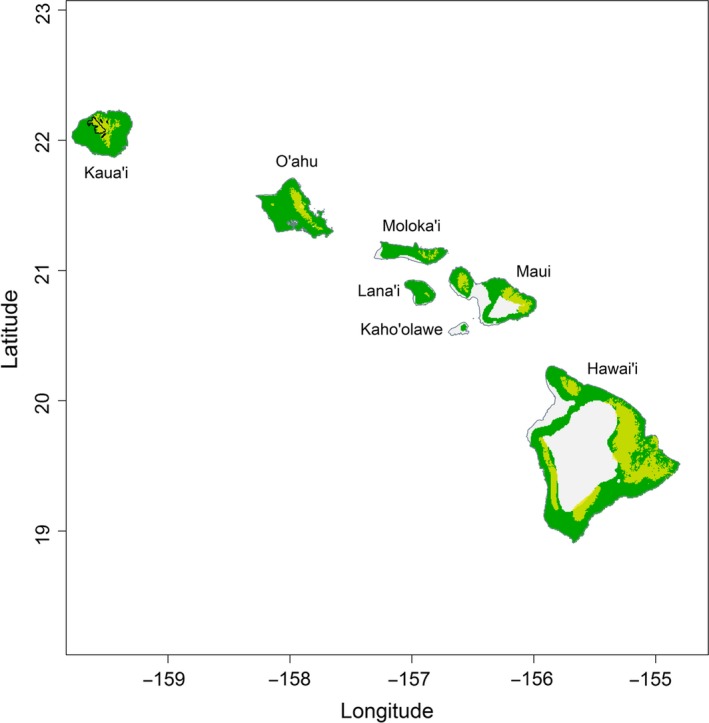
Analogous climate on Kaua'i projected across the other main Hawaiian Islands show where similar climatic conditions currently exist on other neighboring islands. Areas in green highlight locations where baseline climates found on Kaua'i presently exist on other islands. The shaded yellow areas highlight where current compatible forest habitats exists on other islands. The current range of the endemic ‘akeke’e and ‘akikiki, outlined in black, shows that these species only occupy a subset of the entire climatic range and forest habitats on Kaua’i

Projected range responses to climate shifts across the archipelago require transferability, or the ability for models to be projected beyond the settings used for model fitting. The Hawaiian archipelago offers a suitable environment for high model transferability due to the partial replication of wide climate gradients available across all islands. Based on the extreme values of the four selected environmental predictors (Bio1, Bio7, Bio12, and Bio15) on Kaua'i, a rectilinear surface range envelop (SRE) was interpolated using the “biomod2” R package (Thuiller, Engler, 2014) to determine areas of comparable climates within the climatic parameters on other islands. By only selecting four bioclimatic variables and thus refining the complexity of our models, ensemble modeling techniques applied have greater transferability. Analog climates for Kaua'i mapped across the Hawaiian archipelago (Figure 2) show large overlap in climatic conditions among the islands of Kaua'i, Maui, and Hawai'i. While analogous climates do exist on neighboring islands, the endemic ‘akeke’e and ‘akikiki only occupy a small subset of the entire climatic conditions found on Kaua’i. The narrow ranges of these endemic species and the availability of suitable forest habitats limit the total appropriate climate‐based area available across the archipelago. Past studies have shown that species models with good fit generally have higher transferability (Randin et al., 2006; Verbruggen et al., 2013). We evaluated response curves from models to preclude models that had complex responses that would lead to poor transferability and avoid overfitting. We also optimized the boosted regression trees model complexity in terms of number of trees to avoid over fitting to improve transferability. Lastly, we ensured the models behaved in expected ways by projecting distributions on a set of diverse climate scenarios including a “cooling” scenario which showed an expected increase in the range of species at lower elevations, demonstrating our models are not inherently pessimistic or with low transferability. For presence‐only SDM techniques, model transferability improves based on the suitability and relevance of selected predictors (Randin et al., 2006; Vanreusel, Maes, & Van Dyck, 2007). The well‐documented link of temperature and precipitation to avian malaria and consequently native bird distribution (Ahumada et al., 2004; LaPointe, Atkinson, & Samuel, 2012; LaPointe, Goff, & Atkinson, 2010) further ensures high model transferability of our SDMs.

### 2.5 Climatic niche overlap assessment

Baseline and future distributions were also projected for all destination island endemics using the same SDM ensemble approach. ʻAkekeʻe and ʻakikiki were compared to six endemic forest bird species on Maui and nine species endemic to Hawaiʻi. To assess niche overlap between the native forest birds of Kaua'i and resident destination island species, we calculated the comparative pairwise niche overlap metric, *I* (Vorsino et al., 2014; Warren et al., 2008), among species SDM projections. This metric compares both baseline and future potential climate‐based distributions of ‘akeke’e and ‘akikiki to all resident destination island species, yielding values ranging from 0 (no overlap) to 1 (complete overlap). A threshold value greater than 0.8 was selected as the overlap value indicating species niche similarity based on the upper quartile (75% percentile) of *I* value distributions. The *I* statistic was determined to be most appropriate for presence‐only SDM approaches as it compares the suitable climate‐based ranges of species pairs while making no previous biological assumptions about habitat (Vorsino et al., 2014; Warren et al., 2008). Besides the calculated niche overlap values, the physical amount of shared geographic space on destination islands was also compared under current and future climatic scenarios.

## 3 RESULTS

### 3.1 Archipelago‐wide projections

**Figure 3:**
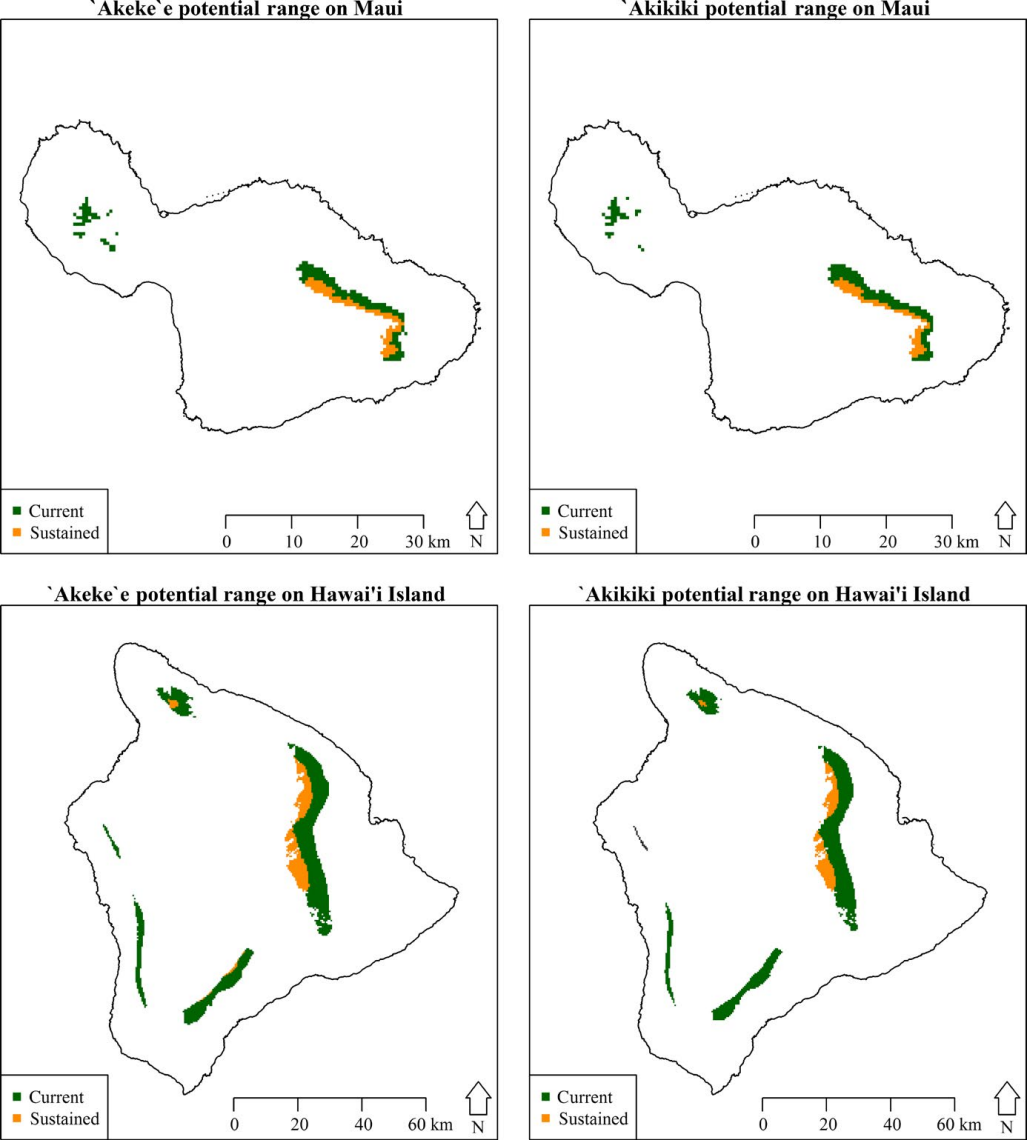
Potential projected current (baseline) and sustained (through end‐of‐century) ranges of Kaua'i endemics ‘akeke’e (*Loxops caeruleirostris*) and ‘akikiki (*Oreomystis bairdi*) on the destination islands of Maui and Hawai’i Island. The sustained areas in yellow reflect the ranges that currently exist and will continue to persist through end‐of‐century

We used our SDM ensemble approach to determine possible baseline and future climate‐based ranges for Kaua'i endemic species on other main Hawaiian Islands. These climate‐based ranges were further limited to areas within suitable vegetation cover to more accurately project where these species could realistically occur. Potential ranges for Kaua'i species were found to exist on Moloka'i, Maui, and Hawai'i under baseline conditions (Table 1). However, only Maui and Hawai'i provide climate‐based ranges for ‘akeke’e and ‘akikiki that persist until end‐of‐century (Figure 3). No suitable ranges were projected to exist on O'ahu, Lana'i, or Kaho’olawe under either baseline or future scenarios. These climatically compatible areas were projected to endure on Maui and Hawai'i under all climate scenarios considered, albeit only in small areas using simplified distribution projections under RCP 8.5 (Fig. S1, Table S2). While both destination islands retain climate‐based ranges for these at‐risk species in the future, all scenarios see a decline of at least 50% total area available per island for these two species by end‐of‐century compared to current, baseline conditions.

**Table 1:** The potential ranges for Kaua'i endemic species ‘akeke’e (*Loxops caeruleirostris*) and ‘akikiki (*Oreomystis bairdi*) on destination islands under baseline conditions and a future moderately warmer and wetter scenario (SRES A1B)

Translocation species	Island (total area in km^2^)^a^	Baseline area (km^2^)	Future area (km^2^)	% change
‘Akeke’e	Kaua'i (1,430)	92.0	0.0	−100.0
	Moloka'i (673)	15.0	0.0	−100.0
	Maui (1,880)	81.8	29.0	−64.5
	Hawai'i (10,430)	775.3	197.3	−74.6
‘Akikiki	Kaua'i (1,430)	63.0	0.0	−100.0
	Moloka'i (673)	7.3	0.0	−100.0
	Maui (1,880)	77.3	25.3	−67.3
	Hawai'i (10,430)	664.8	142.0	−78.6

a No suitable ranges were projected to exist on O'ahu, Lana'i, or Kaho'olawe under either baseline or future conditions.

### 3.2 Climatic niche overlap analysis

**Figure 4:**
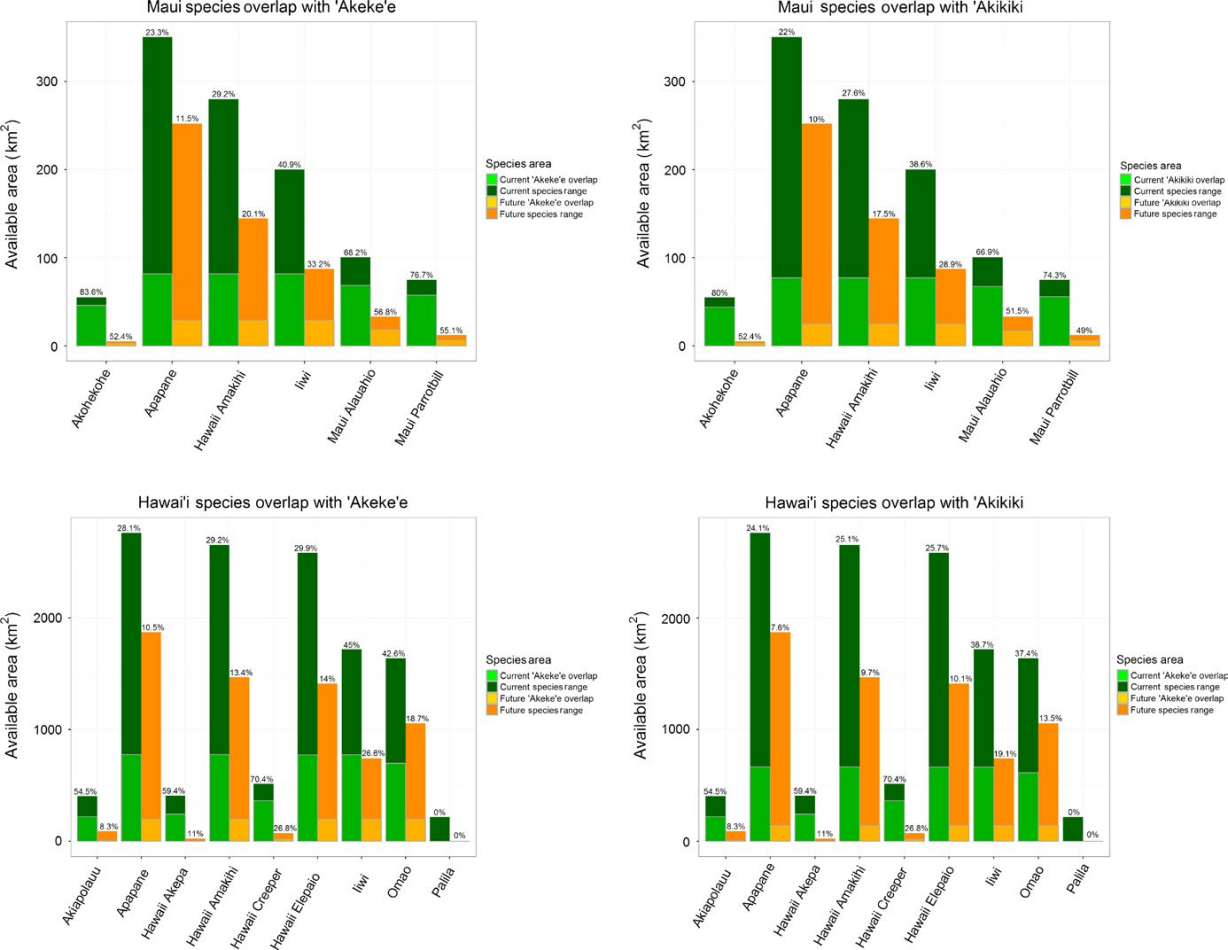
Current and future climate‐based ranges and overlap of Kaua'i ‘akeke’e and ‘akikiki compared to endemic species on potential destination island of Maui and Hawai’i Island. The dark green and orange bars show the amount of area (km^2^) available to the existing island endemics under both current and future projections respectively. The light green and yellow represent the amount of area that overlaps with the projected ranges of ‘akeke’e and ‘akikiki. The percent of overlap amount is indicated by the number above each individual bar

Most niche overlap analyses comparing Maui and Hawai'i native species with the Kaua'i endemics indicate that they occupy distinct climatic space (Table S3). Of the native species on Maui, only the Maui ‘alauahio (*Paroreomyza montana*) was calculated to currently have substantial overlap in climatic niche with ‘akeke’e based on a Warren's *I* value >0.8. On the island of Hawai'i, the Hawai'i creeper (*Oreomystis mana*) was found to currently have high niche overlap (*I *>* *0.8) with both Kaua'i endemic species. The niche overlap metric value derived from the sums of pairwise differences between the two developed SDMs was weakly correlated (*r* ^2^ = .5334) to the amount of geographic area overlapping among species. This is partially expected as the niche overlap metric also accounts for differences in suitability across areas any two species are projected to occur. Comparing the area of overlap to the native species’ ranges on each destination island shows the potential impact on the native avifauna communities. The overlap of shared climate‐based range between the destination island species and the Kaua'i endemic species is estimated to be as high as 70% on Maui and 84% on Hawai'i Island under current climatic conditions and up to 57% on Maui and 52% on Hawai'i based on future projections. However, this shared geographic space is relative to the amount of area available to each individual native species which is projected to shrink under all future climate scenarios considered (Figure 4).

## 4 DISCUSSION

### 4.1 Novelty of methods and approach

The combination of SDM projections of baseline and future distributions, along with a niche overlap analysis, provides a toolset that can be used as the first step to aid conservation decisions regarding the consideration of potential species translocations. These niche overlap values are based solely on climatic space and we realize that a more in‐depth analysis on ecological similarities between different forest bird species would be the next logical step in determining the entire feasibility of translocation. Such further analysis of these characteristics and other factors (Table S4) would determine the ultimate viability of translocation and is beyond the scope of this research. Nevertheless, our methods should be applicable to conservation management for other similarly isolated islands or mountain ranges, making this approach relevant beyond the scope of the Hawaiian Islands. The wide range of climates across the islands, clear climate‐based threats to island endemics, and interisland isolation makes the Hawaiian archipelago well suited for the consideration of translocation and reintroduction options.

Our analysis was performed using various future climate scenarios given the inherent uncertainty of global emissions trajectories. The actual distribution of these species in the future will be dependent on the actual global emissions and associated climate impacts. As our full model projections focus on SRES A1B (a moderate warming scenario), our results indicate that climatically suitable ranges will be likely available for these two species under mild‐to‐moderate future climate shifts on other islands, while the actual pattern of future potential ranges for these Kaua'i forest bird species will be based on the actual future climate scenario that occurs. Our comparison of projected species distribution shifts based on multiple future climate scenarios indicates that realized future warming will be the primary determinant for these species' distributions.

Previous studies that used SDMs to refine reintroduction efforts (Martínez‐Meyer et al., 2006; Pearce & Lindenmayer, 1998; Vitt et al., 2009) focused on areas within the species' previously known historic range. Other studies conducted niche overlap analyses to address habitat changes and degradation (Vorsino, King, Haines, & Rubinoff, 2013; Vorsino et al., 2014; Warren et al., 2008) but have not applied such methods specifically for conservation through translocation. Rather than simply identify and describe suitable areas for translocation, the climate‐focused niche overlap takes into consideration existing native bird species on destination islands. While there are different approaches and other niche analyses that go beyond the scope of this particular analysis, our combined approach offers a wider breadth of information that can be used for considering translocation as a possible and viable conservation option.

Past research on the threat of mosquitos and avian malaria to forest birds has shown that the large distributional shifts detailed in our study are possible (Benning et al., 2002; Liao et al., 2015). However, in attempting to explore the viability of translocation of forest bird species across the Hawaiian archipelago, we did not focus our efforts on modeling the distribution of disease and mosquitoes themselves. Besides the general lack of spatial data on the disease and vector, modeling either of these distributions would not address the differential tolerance of forest bird species to disease nor the differential environmental requirements between vector and disease (LaPointe et al., 2012). Nevertheless, because the ongoing and projected range contractions for these forest bird species are strongly related to a warming‐related shift upslope of avian malaria and its vector, it is important to consider how successful efforts to limit the spread of the disease or vector would impact our findings. Management actions such as traditional vector control (LaPointe, 2008) or novel vector control techniques including sterile mosquito releases could at least partially weaken the strong link between rising temperatures and shrinking forest bird ranges. Additionally, current habitat management activities may foster more robust forest birds populations which have a better chance of evolving increased disease resistance (Kilpatrick, 2006).

### 4.2 Species comparisons beyond climate‐based distributions

While climates analogous to those on Kaua'i can be found across the Hawaiian Islands, other determinants such as the distribution of suitable forest habitats or the interaction with other species may be limiting factors. Therefore, it is important to further evaluate the available habitat ranges for these endemic species of Kaua'i beyond just the projected climate‐based range. The similarity in climate‐based ranges between ‘akeke’e and ‘akikiki is evidenced by their large range overlap and their roughly equivalent niche overlap metrics with Maui and Hawai’i species (Figure 4, Table S3). Despite similar climatic niche characteristics and habitat requirements, these endemic species have divergent foraging and microhabitat uses that allows them to coexist on Kaua'i (Foster, Scott, & Sykes, 2000; Lepson & Pratt, 1997). While ‘akeke’e and ‘akikiki are both insectivores, preferring arthropods such as caterpillars and spiders, their foraging behaviors differ, likely resulting in diets that are drawn from different arthropod communities. Specifically, ‘akeke’e is part of a group of Hawaiian forest bird species that have evolved cross‐bill beaks that help them forage for insects on ‘ohi’a (*Metrosideros polymorpha*) leaf buds. In contrast, ‘akikiki is a creeper, characterized by a generalist bill shape and broader range of foraging substrates which provide a larger range of food resources. Additionally, foraging characteristics, nesting strategy, microhabitat use, and other behaviors presumably contribute to their ability to coexist in similar areas rather than being in direct competition. Niche comparisons among forest bird species are important to understand when considering the potential for competitive exclusion or the ability for stable coexisting populations (Wiens, 1977).

Previous research has shown that although there are considerable niche similarity and phylogenetic proximity of Hawaiian forest birds, there is limited evidence of competition among sympatric species under the theory of competitive exclusion (Mountainspring & Scott, 1985; Scott, Mountainspring, Ramsey, & Kepler, 1986). However, Hawai'i forest birds have evolved and radiated across the islands such that many species belong to sister‐species groups, with a closely related species on each major island (Pratt, Atkinson, Banko, Jacobi, & Woodworth, 2009). For example, the ‘akeke’e belongs to the ‘ākepa group, which once contained four species each distributed on a separate island, including the extant Hawai’i ‘ākepa (*Loxops coccineus*), and the extinct Maui ‘ākepa (*Loxops ochraceus*) and O'ahu ‘ākepa (*Loxops wolstenholmei*). Likewise, the ‘akikiki is very similar to the extant Hawai’i creeper, until recently placed in the same genus *Oreomystis* due to convergent evolution; (Reding, Freed, Cann, & Fleischer, 2010) and the Maui ‘alauahio, as well as the extinct Lāna'i ‘alauahio (*P. montana montana*), kākāwahie (*Paroreomyza flammea*), and O'ahu ‘alauahio (*Paroreomyza maculata*). Thus, allopatric distribution of closely related species has likely minimized competitive exclusion and has not allowed for evidence of such interspecific competition across the Hawaiian archipelago.

Nevertheless, several other factors determining suitability of forest bird translocations remain to be explored. Interactions with non‐native species (Freed & Cann, 2009), and the effects of future potential vegetation changes also have to be investigated (Price et al., 2012) in conjunction with the results provided in this study. While the static vegetation layer used in the analysis may seem arbitrarily conservative, it is not an overly pessimistic assumption. First, vegetation lags to past and ongoing climate shifts have been shown in literature to be in the order of decades (Hughen, Eglinton, Xu, & Makou, 2004; Kitayama, Mueller‐Dombois, & Vitousek, 1995; Wu et al., 2015). Second, mature, structurally complex native forests at high elevations likely take decades to develop given the very slow growth of its dominant species, ‘ohi’a (Atkinson, 1970; Drake & Mueller‐Dombois, 1993). Lastly, the upper limits of forest in high elevation islands in Hawaii are capped by the height and frequency of trade wind inversion (TWI). There is no indication that TWI height is likely to substantially rise in the future and, in fact, a consistent current pattern of increased TWI frequency likely means a lowering of the tree line (Cao, Giambelluca, Stevens, & Schroeder, 2007; Sperling, Washington, & Whittaker, 2004). It is also important to consider the context of past forest bird extinctions. For example, the Maui ‘ākepa, last seen in the 1980s and now presumed extinct, presents both an opportunity and a dilemma. On one hand, translocating ‘akeke’e to Maui could fill an ecological vacuum left by the extinct Maui ‘ākepa, but the reasons that the Maui ‘ākepa went extinct are unknown and perhaps the ‘akeke’e would face a similar fate. Furthermore, as all of these species are currently undergoing population and range declines, the equilibrium distributions of these species are likely smaller than the ranges projected in this study, making accurate site‐specific range predictions particularly challenging. These considerations highlight the complexity and breadth of aspects to consider in developing successful conservation management practices.

### 4.3 Translocation as a conservation option for at‐risk species

Although the projected extirpation of these Kaua'i endemic species is not certain, without additional conservation actions, at‐risk species such as these two have an increased vulnerability to extinction as a result of rapid ongoing and projected climate change (Atkinson et al., 2014; Benning et al., 2002; Fortini et al., 2015; IUCN, 2016b; Liao et al., 2015; Loss, Terwilliger, & Peterson, 2011; Paxton et al., 2016). The potential translocation of threatened species outside their current known range should be comprehensively evaluated as one potential conservation option. While there are drawbacks and risks to translocation, including species competition, becoming invasive, being extirpated from the new area, and hybridization, translocation offers the potential to preserve species that are increasingly vulnerable to extinction and to establish sustainable populations elsewhere. Our maps of prospective areas for translocation are primarily intended to help facilitate a broader discussion of translocation for these at‐risk species. With these results as a starting point, future research should provide a more detailed analysis of niche overlap focused on species for which our analyses indicate a greater similarity in climate‐based ranges. Supplemental information (Tables S5 and S6) provides a brief overview of these additional niche characteristics, such as dietary preferences and nesting habits. Additionally, future research can also better describe the local habitat requirements and competitive interactions among forest bird species at candidate translocation areas identified in our research.

While translocation outside of the known historic range can be controversial, conservation efforts for species like the Laysan Duck (*Anas laysanensis*) (Reynolds, Seavy, Vekasy, Klavitter, & Laniawe, 2008; Reynolds et al., 2013), Nihoa Millerbird (Farmer, Kohley, Freifeld, & Plentovich, 2011; Freifeld et al., 2012), and Hawaiian Monk Seal (*Monachus schauinslandi*) (Baker et al., 2011) provide examples of successful translocations of endemic species already in practice in the Hawaiian Islands. Our results suggest that Kaua'i endemic species may be successfully established on other Hawaiian Islands where suitable climatic and disease‐free space may persist through end‐of‐century. As such, our analysis can be similarly useful for assessing other species groups inhabiting portions of spatially isolated areas, such as other island chains or mountain ranges. As timing is crucial in the conservation of at‐risk species, this study aims to initiate and support the evaluation of the viability of this option for Kaua'i ‘akeke'e, and ‘akikiki. Although not the ultimate solution for conservation of vulnerable species, nor the only option to consider, interisland translocation essentially buys time for species facing the consequences of global warming, especially if these changes continue on the current trajectory through end‐of‐century.

## CONFLICT OF INTEREST

The authors have declared that no competing interests exist.

## AUTHOR CONTRIBUTIONS

All authors focussed on providing scientific research to support and implement conservation management in Hawaii and throughout the Pacific. L.B.F. conceived research idea; L.R.K. conducted simulations and analysis; A.E.V. provided niche overlap methods; L.R.K. and L.B.F. led manuscript writing to which all authors contributed; E.H.P. and J.D.J helped interpret the biological and conservation implications of the findings.
