# Supplementary figures and images for: Assessing the potential of translocating vulnerable forest birds by searching for novel and enduring climatic ranges
Source: Ecol Evol. 2017 Sep 27;7(21):9119–30. doi: 10.1002/ece3.3451 (PMC5677496; doi:10.1002/ece3.3451)

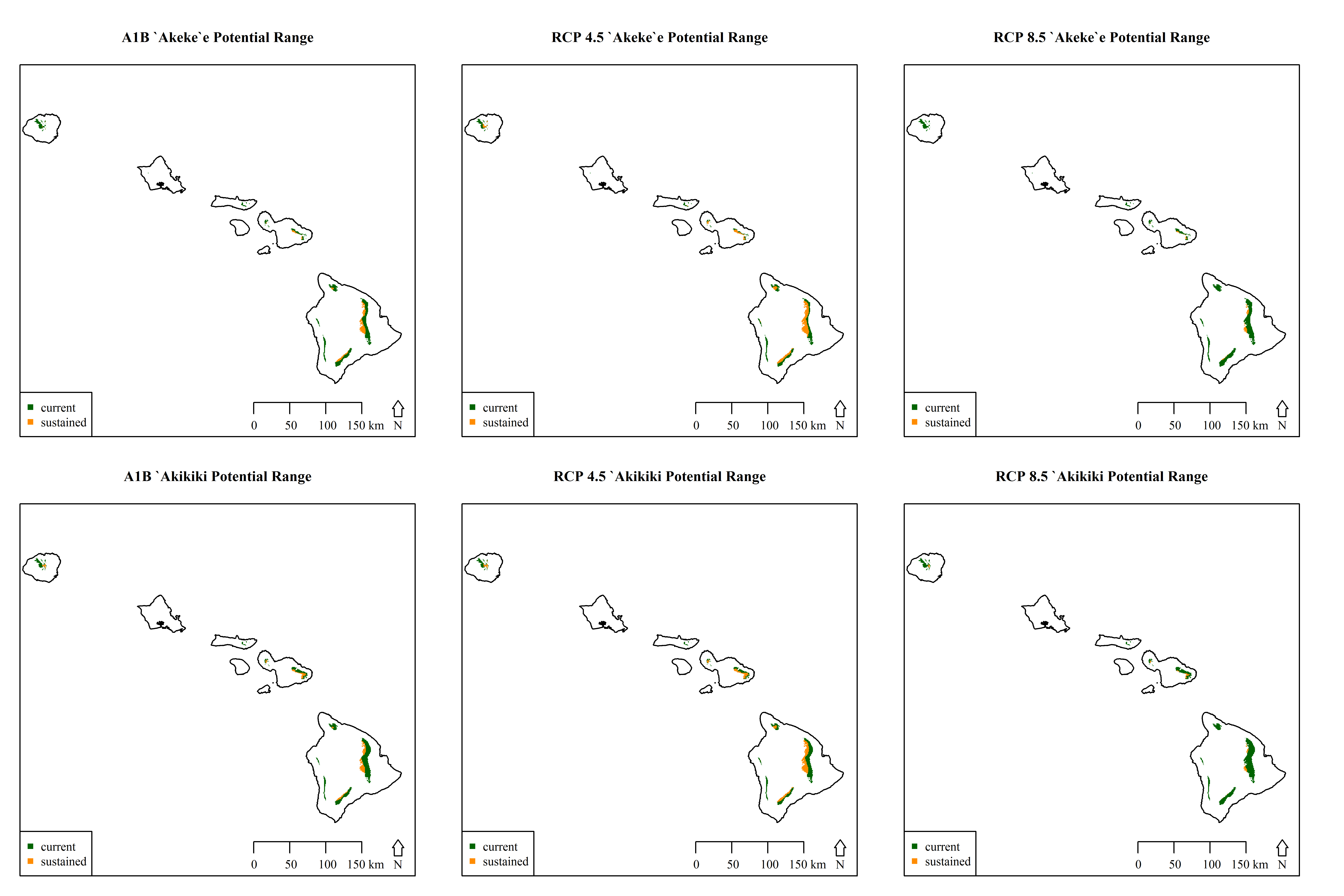

Supplement: Supplementary file 1 [file ECE3-7-9119-s001.tif]
