# Supplementary material for: Assessing the potential of translocating vulnerable forest birds by searching for novel and enduring climatic ranges
Source: Ecol Evol. 2017 Sep 27;7(21):9119–30. doi: 10.1002/ece3.3451 (PMC5677496; doi:10.1002/ece3.3451)
Supplement: Supplementary file 2 [file ECE3-7-9119-s002.docx]

# SUPPORTING INFORMATION

**Table S1** Comparison of the Receiver Operating Characteristic (ROC) scores used to evaluate species distribution models.

| Climate Scenario | Bioclimatic Variables | Endemic Species | Mean ROC |
| --- | --- | --- | --- |
| SRES A1B | Bio1, Bio7  Bio12, Bio15 | ‘Akeke’e  ‘Akikiki | 0.9769  0.9804 |
| SRES A1B  (*simplified*) | Bio1, Bio12 | ‘Akeke’e  ‘Akikiki | 0.9835  0.9807 |

**Table S2** Calculated available range area based on simplified model results for Kaua’i endemic ‘akeke’e (*Loxops caeruleirostris*) and ‘akikiki (*Oreomystis bairdi*) species.

| Translocation species | Climate scenario ^a^ | Baseline area (km^2^) ^b^ | Future area (km^2^) ^b^ | % Change ^c^ |
| --- | --- | --- | --- | --- |
| ‘Akeke’e | SRES A1B | 907.00 | 215.25 | -76.27 |
|  | RCP 4.5 | 907.00 | 415.25 | -54.23 |
|  | RCP 8.5 | 907.00 | 73.25 | -91.92 |
| ‘Akikiki | SRES A1B | 1026.25 | 229.50 | -77.64 |
|  | RCP 4.5 | 1026.25 | 383.25 | -62.66 |
|  | RCP 8.5 | 1026.25 | 65.00 | -93.67 |

^a^ Different emissions scenarios run to compare simplified model results

^b^ Amount of range area (km^2^) available for Kaua’i endemics across the Hawaiian archipelago for baseline and future projections

^c^ Difference in amount of change (%) in range available

**Table S3** Climate-based range similarities and overlap of Kaua’i ‘akeke’e and ‘akikiki and island endemic species present on the potential destination islands of Maui and Hawai’i Island.

| Translocation species | Destination island | Destination island species | Baseline niche equivalency ^a^ | % Range overlap baseline ^c^ |
| --- | --- | --- | --- | --- |
| ‘Akeke’e | Maui | ‘Ākohekohe  (*Palmeria dolei*) | 0.77 | 83.64 |
|  |  | ‘Apapane  (*Himatione s. sanguinea*) | 0.5 | 23.32 |
|  |  | Hawai’i ‘Amakihi  (*Hemignathus virens*) | 0.52 | 29.20 |
|  |  | ‘I’iwi  (*Vestiaria coccinea*) | 0.58 | 40.88 |
|  |  | Maui ‘Alauahio  (*Paroreomyza flammea*) | *0.82 ^b^ | 68.16 |
|  |  | Maui Parrotbill  (*Pseudonestor xanthophrys*) | 0.61 | 76.67 |
| ‘Akeke’e | Hawai’i | ‘Akiapōlā’au  (*Hemignathus munroi*) | 0.76 | 54.47 |
|  |  | ‘Apapane | 0.51 | 28.09 |
|  |  | Hawai’i ‘Ākepa  (*Loxops caeruleirostris*) | 0.73 | 59.42 |
|  |  | Hawai’i ‘Amakihi | 0.5 | 29.22 |
|  |  | Hawai’i Creeper  (*Oreomystis mana*) | *0.83 | 70.44 |
|  |  | Hawai’i ‘Elepaio  (*Chasiempis sandwichensis*) | 0.68 | 29.93 |
|  |  | ‘I’iwi | 0.58 | 45.05 |
|  |  | ‘Ōma’o  (*Mysdestes obscurus*) | 0.67 | 42.64 |
|  |  | Palila  (*Loxioides bailleui*) | 0.59 | 00.00 |
| ‘Akikiki | Maui | ‘Ākohekohe | 0.75 | 80.00 |
|  |  | ‘Apapane | 0.49 | 22.04 |
|  |  | Hawai’i ‘Amakihi | 0.47 | 27.59 |
|  |  | ‘I’iwi | 0.56 | 38.63 |
|  |  | Maui ‘Alauahio | 0.78 | 66.92 |
|  |  | Maui Parrotbill | 0.70 | 74.33 |
| ‘Akikiki | Hawai’i | ‘Akiapōlā’au | 0.77 | 54.47 |
|  |  | ‘Apapane | 0.48 | 24.09 |
|  |  | Hawai’i ‘Ākepa | *0.81 | 59.42 |
|  |  | Hawai’i ‘Amakihi | 0.47 | 25.07 |
|  |  | Hawai’i Creeper | *0.90 | 70.44 |
|  |  | Hawai’i ‘Elepaio | 0.74 | 25.70 |
|  |  | ‘I’iwi | 0.57 | 38.68 |
|  |  | ‘Ōma’o | 0.73 | 37.37 |
|  |  | Palila | 0.51 | 00.00 |

^a^ Climatic niche equivalency values based on Warren’s *I* values range from 0 (no overlap) to 1 (complete overlap)

^b^ High niche equivalency values above 0.8 threshold denoted by (*)

^c^ Percent (%) overlap indicates how much range is potentially shared between species pairs based on projected distributions

**Table S4** Detailed ecological characteristics and behaviors among native Hawaiian forest birds of Kaua’i, [K], Maui [M], and Hawai’i Island [H]. (*Source:* Adapted from Pratt et al. 2009).

Endemic Kaua’i [K] Forest Birds

| Food  Guild ^a^ | Degree of Specialization ^b^ | Historic  Habitats ^c^ | Habitat or Host Plant Type ^d^ | Foraging Substrate ^e^ | Foraging Behavior ^f^ | Dietary Preferences ^g^ |
| --- | --- | --- | --- | --- | --- | --- |
| ‘Akeke’e (*Loxops caeruleirostris*) [K] | | | | | | |
| A | S | LM, LW, MM, MW | ‘Ōhi’a forests | Outer branches, terminal leaf canopy | Pry open leaf buds, silk-bound leaves for arthropods | Caterpillars, insect eggs, spiders |
| ‘Akikiki (*Oreomystis bairdi*) [K] | | | | | | |
| A | I | LD, LM, LW, MM, MW | ‘Ōhi’a forests | Bark, dead wood, and foliage of trees and subcanopy | Glean, probe for arthropods while creeping and clinging | Caterpillars, moths, beetles, spiders |

^a^ Food guild based on most frequently obtained or typical foods consumed by species

A – arthropods (including snails); F&S – fruits and seeds; N – nectar; R – raptorial

^b^ Degree of specialization from restricted niche breadth of species based on limits of habitat and diet

G – generalist; I – intermediate; S – specialist

^c^ Historic habitat based on vegetation classifications system and types for Hawai**’**i (from Scott et al. 1986; Pratt and Gon 1998)

CD – coastal dry shrubland; LD – lowland dry forest; LM – lowland mesic forest; LW – lowland wet forest; MD – montane dry forest; MM – montane mesic forest; MW – montane wet forest; SD – subalpine dry woodland; AD – alpine dry shrubland

^d^ Habitat or host plant type of environment where species naturally tend to occur (from Birds of North America accounts)

^e^ Foraging substrate based on natural surface where species search for food (from Birds of North America accounts)

^f^ Foraging behavior based on manner or methods in which species search for food (from Birds of North America accounts)

^g^ Diet based on typical food selection of species (from Birds of North America accounts)

Maui [M] and Hawai’i Island [H] Forest Birds

| Name *(*Family**, Species)* [Island] | | | | | | |
| --- | --- | --- | --- | --- | --- | --- |
| Food  Guild | Degree of Specialization | Historic  Habitats | Habitat or Host Plant Type | Foraging Substrate | Foraging Behavior | Dietary Preferences |
| ‘Akiapōlā’au *(Hemignathus munroi)* [H] | | | | | | |
| A | S | LD, LM, LW, MD, MM, MW, SD | Koa, koa-’ōhi’a, ‘ōhi’a, and māmane forests | Branches, naio, trunks, māmane, kolea, ‘ōhi’a, ‘ie’ie vines | Creeps, hammers, probes, and hooks wood | Beetles, caterpillars, spiders, ‘ōhi’a sap |
| ‘Ākohekohe *(Palmeria dolei)* [M] | | | | | | |
| N | I | LM, MW | ‘Ōhi’a forests | Middle/upper canopy on terminal branches | Probe, nectar-rob, glean, and defend nectar resources | ‘Ōhi’a nectar, caterpillars, flies, spiders |
| ‘Apapane *(Himatione s. sanguinea)* [H, M] | | | | | | |
| N | G | LD, LM, LW, MD, MM, MW, SD, AD | ‘Ōhi’a, koa forests | Middle/upper canopy on terminal branches | Probe, glean, and track nectar sources | ‘Ōhi’a and māmane nectar, insects, spiders |
| Hawai’i ‘Ākepa *(Loxops coccineus)* [H] | | | | | | |
| A | S | LM, LW, MM, MW | ‘Ōhi’a, koa forests | Terminal leaf cluster and small branches | Pry open buds and leaves for arthropods | Caterpillars, insects, insect eggs, spiders |
| Hawai’i ‘Amakihi *(Hemignathus virens)* [H, M] | | | | | | |
| A | G | LD, LM, LW, MD, MM, MW, SD, AD | Wide variety of forests and shrubs | Terminal branches of trees, shrubs, and vines | Glean for arthropods, probe and nectar-rob | Insects, ‘ōhi’a and māmane, nectar, small fruits, juices |
| Hawai’i Creeper *(Oreomystis mana)* [H] | | | | | | |
| A | I | LM, LW, MM, MW, SD | ‘Ōhi’a, koa forests | Bark, dead wood, and foliage on trunks and branches | Glean, probe for arthropods while creeping and clinging | Caterpillars, moths, beetles, spiders, insect and spider eggs |
| Hawai’i ‘Elepaio *(*Monarchidae, *Chasiempis sandwichensis)* [H] | | | | | | |
| A | G | LD, LM, LW, MD, MM, MW, SD | ‘Ōhi’a forests, variety of vegetation types | Branches, foliage, rotten wood, around trees on ground | Branch and flight glean, aerial fly-catch | Spiders, insects, millipedes, centipedes, slugs |
| ‘I’iwi *(Vestiaria coccinea)* [H, M] | | | | | | |
| N | I | LD, LM, LW, MD, MM, MW, SD | ‘Ōhi’a, koa forests | Middle/upper canopy, vines, branches, understory | Probe, nectar-rob, glean, track and defend nectar sources | ‘Ōhi’a, māmane, and lobelia nectar, insects, spiders |
| Maui ‘Alauahio *(Paroreomyza flammea)* [M] | | | | | | |
| A | I | LD, LM, LW, MM, MW | ‘Ōhi’a forests, māmane savannah, shrubland | Bark, dead wood, and foliage on trunks and branches | Glean and probe for arthropods | Caterpillars, moths, beetles, spiders, insect and spider eggs |
| Maui Parrotbill *(Pseudonestor xanthophyrs)* [M] | | | | | | |
| A | S | LD, LM, LW, MM, MW | ‘Ōhi’a, koa forests | Branches of trees and shrubs | Bite, split, and pry wood and fruits, glean branches | Beetle larvae, pupae caterpillars |
| ‘Ōma’o *(*Turdidae*, Myadestes obscurus)* [H] | | | | | | |
| F & S | G | LD, LM, LW, MD, MM, MW, SD, AD | Closed and open forests, dense vegetated valleys, streams | Vertical range of forest canopy, shrubs, epiphytes | Plucks, eats, and swallows fruits, gleans, probes, and scratches | Small/medium fruits, insects, spiders |
| Palila *(Loxioides bailleui)* [H] | | | | | | |
| F & S | S | LD, MD, MM, SD | Māmane, naio | Terminal branches in large to medium tree canopies | Pick and shred pods and buds, nectar-rob, glean branches | Māmane seeds, glower parts, nectar, leaves, caterpillars |

* Of the family Fringillidae and subfamily of Drepanidinae unless otherwise noted

**Table S5** Thresholds used to categorize species similarity in species characteristics among the considered translocation species of Kaua’i and the endemic species of Maui and Hawai’i Island. The additional characteristics beyond the niche overlap analysis considered were assessed based on the information about each species found in the corresponding ‘Source’ column. By conducting a thorough literature review of these sources per species, different levels of comparison were developed based on the variety of descriptions detailed for each category.

| Category | Description | HIGH | MED | LOW | Source |
| --- | --- | --- | --- | --- | --- |
| Baseline niche overlap | calculated baseline Warren's *I* value | *I* > 0.8 | 0.8 > *I* > 0.6 | *I* < 0.6 | niche overlap analysis |
| Future  niche overlap | calculated future Warren's *I* value | *I* > 0.8 | 0.8 > *I* > 0.6 | *I* < 0.6 | niche overlap analysis |
| Phylogenetic proximity ^a^ | evolutionary divergence and history | < 2 levels of divergence | 2 < divergence levels < 6 | > 6 levels of divergence | Lerner et al. 2011 |
| Dietary niche similarity ^b^ | food guilds and degree of specialization | same food guild  AND specialization | same food guild  OR specialization | different food guild  AND specialization | Pratt et al. 2009 |
| Nesting niche similarity ^c^ | nest dimensions, location, composition… | > 4 shared characteristics | 3-4 shared  characteristics | < 3 shared characteristics | BNA Online 2015 |

^a^ Individual species comparisons based on the species evolutionary divergence over time where species more closely related historical have a higher degree of similarity while species with greater divergence are categorized to have lower phylogenetic proximity

^b^ Species with the same specialization (generalist, intermediate, specialist) and within the same food guild (arthropods, fruits and seeds, nectar, raptorial) were considered to be highly similar compared to those species of different specialization and food guilds

^c^ Nesting similarities based on the number of shared nest characteristics as described in each species account where a greater number of shared characteristics (i.e. nest size, composition, build season, etc.) among species were classified as high similarity

**Table S6** Ranked niche characteristics of ‘akeke’e and ‘akikiki compared to destination island endemics of Maui and Hawai’i Island where potential suitable range exist for the species between now and end-of-century. The niche overlap values are based on the calculated Warren’s I values for both baseline and future scenarios. Species’ phylogenetic proximity uses the adaptive radiation of the forest birds as a level of measurement. Dietary habits are based on the food guild and specialization of a species while the nesting habits focus on the historical habitat region preferred by each species. These factors were averaged overall to determine the level of similarity (low – 1, medium – 2, high – 3) among the native Hawaiian forest birds and the Kauaʻi endemic species. Averaged ranks of 2.4 or higher are considered to have greater overlap (see Appendix S6 for additional ranking information).

| Translocation species | Destination island | Destination  island species | Baseline  niche overlap ^b^ | Future  niche overlap | Phylogenetic proximity ^c^ | Dietary niche similarity ^d^ | Nesting similarity ^e^ | **AVERAGE** |
| --- | --- | --- | --- | --- | --- | --- | --- | --- |
| ‘Akeke’e | Maui | ‘Ākohekohe | 2 ^a^ | 1 | 1 | 1 | 1 | **1.2** |
|  |  | ‘Apapane | 1 | 1 | 1 | 1 | 2 | **1.2** |
|  |  | Hawai’i ‘Amakihi | 1 | 1 | 2 | 2 | 2 | **1.6** |
|  |  | ‘I’iwi | 1 | 2 | 2 | 1 | 3 | **1.8** |
|  |  | Maui ‘Alauahio | 3 | 2 | 1 | 2 | 2 | **2.0** |
|  |  | Maui Parrotbill | 1 | 1 | 2 | 3 | 3 | **2.0** |
| ‘Akeke’e | Hawai’i | ‘Akiapōlā’au | 2 | 3 | 2 | 3 | 1 | **2.2** |
|  |  | ‘Apapane | 1 | 1 | 1 | 1 | 2 | **1.2** |
|  |  | Hawai’i ‘Ākepa | 2 | 3 | 3 | 3 | 3 | ***2.8** ^f^ |
|  |  | Hawai’i ‘Amakihi | 1 | 1 | 2 | 2 | 2 | **1.6** |
|  |  | Hawai’i Creeper | 3 | 3 | 3 | 2 | 2 | ***2.6** |
|  |  | Hawai’i ‘Elepaio | 2 | 2 | 1 | 2 | 3 | **2.0** |
|  |  | ‘I’iwi | 2 | 2 | 2 | 1 | 3 | **2.0** |
|  |  | ‘Ōma’o | 2 | 2 | 1 | 1 | 2 | **1.6** |
|  |  | Palila | 1 | 1 | 1 | 2 | 1 | **1.2** |
| ‘Akikiki | Maui | ‘Ākohekohe | 2 | 1 | 2 | 2 | 1 | **1.6** |
|  |  | ‘Apapane | 1 | 1 | 2 | 1 | 3 | **1.6** |
|  |  | Hawai’i ‘Amakihi | 1 | 1 | 1 | 2 | 2 | **1.4** |
|  |  | ‘I’iwi | 1 | 2 | 2 | 2 | 3 | **2.0** |
|  |  | Maui ‘Alauahio | 2 | 2 | 3 | 3 | 3 | **2.6** |
|  |  | Maui Parrotbill | 2 | 1 | 2 | 2 | 1 | **1.6** |
| ‘Akikiki | Hawai’i | ‘Akiapōlā’au | 2 | 3 | 2 | 2 | 1 | **2.0** |
|  |  | ‘Apapane | 1 | 1 | 2 | 1 | 3 | **1.6** |
|  |  | Hawai’i ‘Ākepa | 3 | 3 | 1 | 2 | 3 | ***2.4** |
|  |  | Hawai’i ‘Amakihi | 1 | 1 | 1 | 2 | 2 | **1.4** |
|  |  | Hawai’i Creeper | 3 | 3 | 1 | 3 | 1 | **2.2** |
|  |  | Hawai’i ‘Elepaio | 2 | 2 | 1 | 2 | 3 | **2.0** |
|  |  | ‘I’iwi | 1 | 2 | 2 | 2 | 3 | **2.0** |
|  |  | ‘Ōma’o | 2 | 2 | 1 | 1 | 1 | **1.4** |
|  |  | Palila | 1 | 1 | 2 | 1 | 1 | **1.2** |

^a^ All factors indicate level of similarity among species (low = 1, medium = 2, high = 3)

^b^ Niche overlap values based on Warren’s *I* values for both baseline and future projections

^c^ Species’ phylogenetic proximity based on adaptive radiation of forest birds as a level of measurement

^d^ Dietary habits based on food guild and specialization of species

^e^ Nesting habits based on preferred historical habitat region of species

^f^ High niche equivalency values above 2.4 threshold denoted by (*)
